# Moderate Genetic Diversity and Genetic Differentiation in the Relict Tree *Liquidambar formosana* Hance Revealed by Genic Simple Sequence Repeat Markers

**DOI:** 10.3389/fpls.2016.01411

**Published:** 2016-09-21

**Authors:** Rongxi Sun, Furong Lin, Ping Huang, Yongqi Zheng

**Affiliations:** State Key Laboratory of Tree Genetics and Breeding, Key Laboratory of Silviculture of the State Forestry Administration, Research Institute of Forestry, Chinese Academy of ForestryBeijing, China

**Keywords:** *Liquidambar formosana*, genetic diversity, population structure, genotyping, SSR markers

## Abstract

Chinese sweetgum (*Liquidambar formosana*) is a relatively fast-growing ecological pioneer species. It is widely used for multiple purposes. To assess the genetic diversity and genetic differentiation of the species, genic SSR markers were mined from transcriptome data for subsequent analysis of the genetic diversity and population structure of natural populations. A total of 10645 potential genic SSR loci were identified in 80482 unigenes. The average frequency was one SSR per 5.12 kb, and the dinucleotide unit was the most abundant motif. A total of 67 alleles were found, with a mean of 6.091 alleles per locus and a mean polymorphism information content of 0.390. Moreover, the species exhibited a relatively moderate level of genetic diversity (*He* = 0.399), with the highest was found in population XY (*He* = 0.469). At the regional level, the southwestern region displayed the highest genetic diversity (*He* = 0.435) and the largest number of private alleles (n = 5), which indicated that the Southwestern region may be the diversity hot spot of *L. formosana.* The AMOVA results showed that variation within populations (94.02%) was significantly higher than among populations (5.98%), which was in agreement with the coefficient of genetic differentiation (*Fst* = 0.076). According to the UPGMA analysis and principal coordinate analysis and confirmed by the assignment test, 25 populations could be divided into three groups, and there were different degrees of introgression among populations. No correlation was found between genetic distance and geographic distance (P > 0.05). These results provided further evidence that geographic isolation was not the primary factor leading to the moderate genetic differentiation of *L. formosana*. As most of the genetic diversity of *L. formosana* exists among individuals within a population, individual plant selection would be an effective way to use natural variation in genetic improvement programs. This would be helpful to not only protect the genetic resources but also attain effective management and exploit genetic resources.

## Introduction

Chinese sweetgum (*Liquidambar formosana* Hance) belongs to the genus *Liquidambar* of family Altingiaceae (Santamour, 1972; Bremer et al., 2009). The genus *Liquidambar* is a relic of Tertiary floras and is distributed disjunctively in East Asia, Turkey, and North America (Li et al., 1997). These disjunctive distributions are remnants of their wide distribution during the Tertiary period. This genus had flourished well in a wide area covering East Asia, Central Asia, Asia Minor, America and Central Europe during the Miocene, and disappeared in Europe and Northwest America in the Pleistocene as a result of extensive glaciations (Öztürk et al., 2008). After these glaciations, the natural distributions of *Liquidambar* species were forced into refugia in East Asia, Turkey and North America (Ozdilek et al., 2012). This genus has four main species, including *L. formosana, L. acalycina, L. styraciflua*, and *L. orientalis*. The fossils of *Liquidambar* have been found in many Cenozoic deposits around the world, particularly in Neogene strata in the USA and China; however, the scope of its distribution began to shrink in the late Cenozoic (Li et al., 1984). According to molecular evidence, *L. formosana* and *L. acalycina* comprised a special clade in the phylogeny of the genus *Liquidambar*; the other clade consisted of *L. orientalis* and *L. styraciflua* (Li et al., 1997).

Chinese sweetgum is a large deciduous tree species. The species can reach 40 m in height and 100 cm in diameter. It is naturally distributed across Southern China, north from the Qinling and Dabie Mountains, west to Sichuan and Guizhou, south to Hainan, and east to Taiwan. As a fast-growing pioneer species, it has been widely used for timber production, urban landscaping, and medicinal and ornamental purposes. It produces traumatic gum when its trunk is wounded, and the gum has long been studied for its medicinal and cosmetic applications (Zheng et al., 2015). Trees of the species contain chemical constituents, such as flavonoids, tannins and essential oils, which have pharmacological activities in the treatment of dysentery, rheumatism, tumors, and arrhythmias (Chen et al., 2011; Enriquez et al., 2013).

*Liquidambar formosana* is a Tertiary relict plant and has a long evolutionary history (Kuprianova, 1960). It experienced several glaciations and associated cooling and survived in the south of China but disappeared in the north of China after the Pleistocene glaciations. In recent years, relict plants have received increasing attention from the research community, becoming one of the hotspots of biodiversity conservation and research (Root et al., 2003; López-Pujol et al., 2006). The subtropical region of China is abundant in Tertiary relic plants and was a Quaternary glacial refugia, serving as an important source of plant dispersion post-glaciation (Wu, 1980).

For most perennial tree species, the development of forest genetic resources is a slow process. The lack of genome information and effective molecular markers may be one of the major barriers. Only a few studies have reported on the genetic diversity of *L. formosana* using isozyme and randomly amplified markers systems (Bi et al., 2010; Chai et al., 2013). Currently, with the development of the next generation sequencing (NGS), a large number of genomic and transcriptome data have been published. These data have provided a new tool for the genotyping and assessment of genetic resources in non-model species (Davey et al., 2011; Ekblom and Galindo, 2011; Lin et al., 2011). Moreover, the process and analytic power required to handle the huge sequencing data have improved (Aflitos et al., 2015; Ren et al., 2015). Therefore, the development of a reliable and effective molecular marker system from sequencing data has become feasible in many non-model plants (Durand et al., 2010; Yadav et al., 2011). Many molecular markers have been developed; however, simple sequence repeats (SSR) are more efficient due to their codominant inheritance, high reproducibility, relatively abundance, and numbers of polymorphisms (Powell et al., 1996), which indicate its usefulness in the evaluation of genetic diversity and population structure (Zeng et al., 2010; Zhang et al., 2012; Molosiwa et al., 2015). Furthermore, information revealed by SSR markers is also useful for understanding the patterns of genetic diversity allocated within and among populations, which is particularly important for developing genetic resources conservation and management strategies (Maguire et al., 2002). Indeed, genetic diversity is a prerequisite for species adaptability and evolution (Reed and Frankham, 2003).

We hypothesized that as the result of interactions between glaciations and anthropogenic activities, the genetic diversity of Chinese sweetgum had declined in the past. To test this hypothesis, we developed genic SSR markers based on transcriptome data (Wen et al., 2015) to examine the genetic diversity and structure of Chinese sweetgum populations. We addressed the following specific issues: (1) the genetic diversity and patterns of allocation among and within populations; (2) the genetic differentiation of the population; and (3) the correlation between the genetic distance and geographic distance of *L. formosana*.

## Materials and methods

### Plant materials and DNA extraction

**Figure 1:**
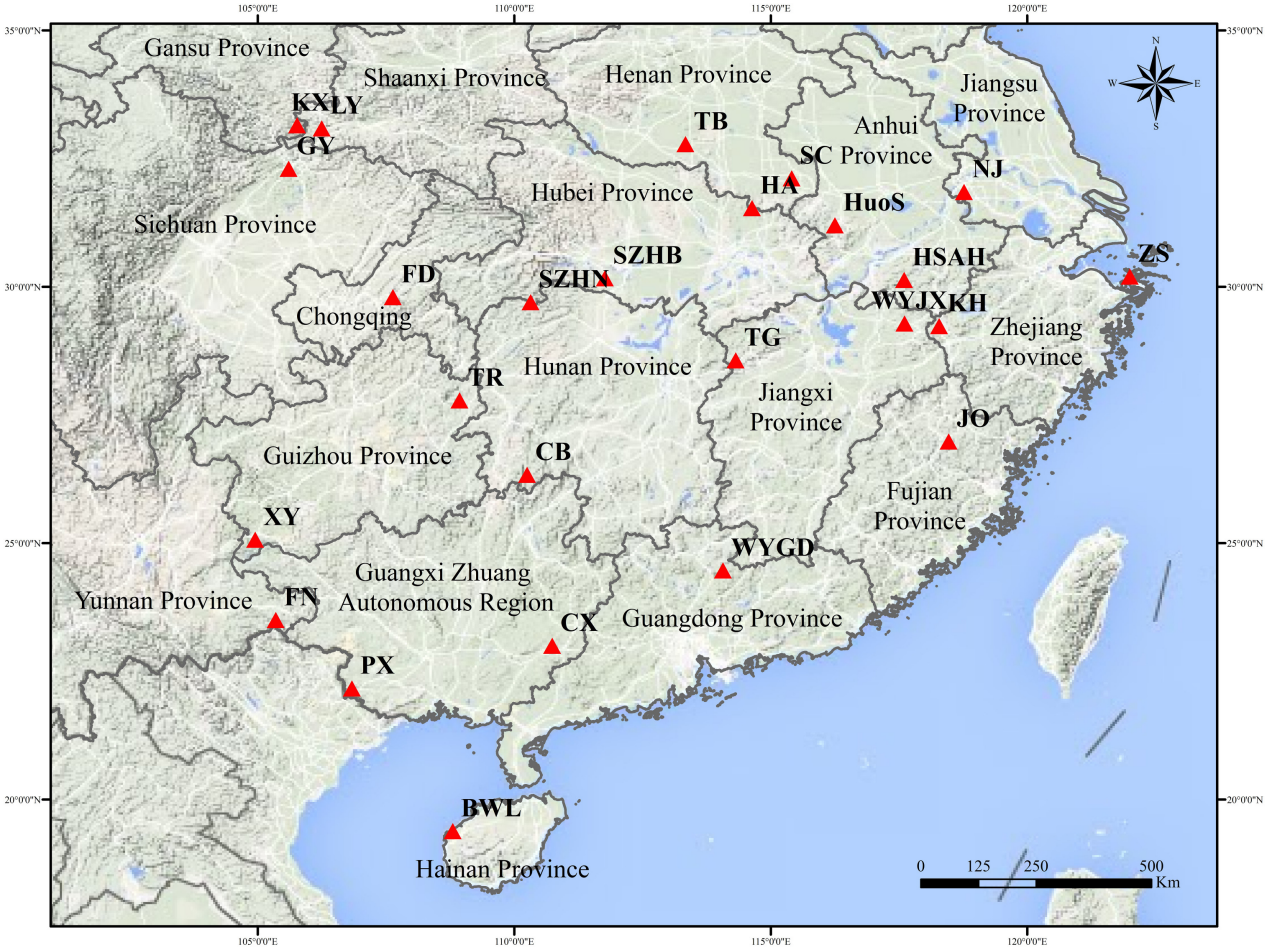
**Sampling distribution of *L. formosana* in China**.

A total of 691 individual trees were sampled from 25 populations across nearly the entire natural range of *L. formosana* in China (Figure 1, Table 1). Each population was represented by 17 to 32 individuals, and each of the sampled individuals was kept more than 50 m apart to minimize the genetic relationships among the sample trees. The 25 populations could be grouped into four regions: Southwestern China, the Dabie Mountains and Foothills, the Coastal region, and the Central region. Fresh young leaves were collected from the sample trees, and the leaf samples were then dried using silica gel and stored in a refrigerator at −80°C for DNA extraction. Total genomic DNA was extracted from dried leaf samples using the Plant Genomic DNA Kit (TIANGEN, Beijing, China). The quality and concentration of extracted DNA were measured by electrophoresis on 0.8% agarose gels and a Microplate Spectrophotometer (Molecular Device, Sunnyvale, CA, USA), respectively. The DNA was diluted to a working concentration of 50 ng/μL for later experiments.

**Table 1:** **Sampling information of *L. formosana***.

**Population code**	**Sampling location**	**Number**	**Longitude (E)**	**Latitude (N)**	**Altitude (m)**
BWL	Bawangling, Hainan	30	108°48′	18°30′	655
PX	Pingxiang, Guangxi	30	106°50′	22°10′	420
TR	Tongren, Guizhou	30	108°56′	27°47′	400
XY	Xingyi, Guizhou	32	104°57′	25°04′	1100
HSAH	Huangshan, Anhui	30	117°36′	30°08′	150
KX	Kangxian, Gansu	29	105°46′	33°09′	1020
LY	Lueyang, Shaanxi	21	105°50′	32°20′	700
TG	Tonggu, Jiangxi	29	114°19′	28°34′	550
WYJX	Wuyuan, Jiangxi	26	117°53′	30°02′	831
CB	Chengbu, Hunan	26	110°15′	26°20′	610
KH	Kaihua, Zhejiang	23	118°17′	29°14′	1453
WYGD	Wengyuan, Guangdong	28	114°04′	24°28′	215
FN	Funing, Yunnan	29	105°21′	23°30′	1100
FD	Fengdu, Chongqing	24	107°38′	29°48′	560
JO	Jianou, Fujian	30	118°28′	26°59′'	204
ZS	Zhoushan, Zhejiang	32	121°60′	30°12′	350
SZHN	Sangzhi, Hunan	26	110°19′	29°42′	700
SZHB	Songzi, Hubei	28	111°46′	30°10′	424
NJ	Nanjing, Jiangsu	24	118°46′	31°51′	28
GY	Guangyuan, Sichuan	29	105°36′	32°18′	890
CX	Cenxi, Guangxi	17	111°10′	22°40′	230
HuoS	Huoshan, Anhui	31	116°15′	31°12′	150
SC	Shangcheng, Henan	30	115°32′	31°44′	410
TB	Tongbai, Henan	27	113°21′	32°21′	300
HA	Hongan, Hubei	30	114° 38′	31°32′	300

### Mining and identification of genic SSR markers

Genic SSR loci were derived from transcriptome data in the National Center for Biotechnology Information (NCBI) database. A short read archive under accession numbers SRR1514949 and SRR1514913 was assembled using Trinity software (Grabherr et al., 2011), and then screened for genic SSR loci from the Unigene library with the by MIcroSAtellite identification (MISA) tool (Thiel et al., 2003). All newly identified SSR sequences have been deposited in GenBank under accession numbers KU356822- KU356835. The gene coding structure was detected by a Transdecoder module of Trinity (Zhao et al., 2011), which showed loci located in 5′ untranslated regions (5′ UTR), 3′ untranslated regions (3′ UTR) or coding sequence (CDS) regions. For SSR identification, the minimum motif repeats were defined as 6 repeats for a dinucleotide unit and 5 repeats for trinucleotide, tetranucleotide, pentanucleotide, and hexanucleotide units: the mononucleotide unit was not included in the SSR search criteria. Primer pairs flanking the SSRs were designed using Primer3 (Koressaar and Remm, 2007) following the core criteria: the optimum length of the primers was 20 bp, ranging from 18 to 22 bp; the annealing temperature was between 55° and 62°C with an optimum annealing temperature of 60°C; the GC content ranged from 40 to 60% with 50% as the optimum; and the optimum size of the PCR product was 100–300 bp. A total of 72 genic SSR primer pairs were designed with the criteria mentioned above. All the designed primers were screened on eight samples, which were chosen at random. The primers producing clear and polymorphic bands were subsequently used for genetic diversity assessments.

### Polymerase chain reaction (PCR) amplifications and genotyping

PCR amplifications were performed in 25 μL reaction volumes as follows: 12.5 μL of 2 × Taq MasterMix (Aid Lab, Beijing, China), approximately 50 ng of DNA, 5 pmol reverse primer and 5 pmol forward primer with the 5′ end labeled with fluorescent dyes (FAM, HEX, or ROX; Ruibiotech, Beijing, China) and sterile double-distilled water added to 25 μL. The amplifications were performed with a touchdown profile, with the first denaturation at 94°C for 5 min, followed by 10 cycles of denaturation for 30 s at 94°C, annealing for 30 s at 63°C and then extension for 45 s at 72°C, with a 1°C decrease in the annealing temperature each cycle; the following 20 cycles included denaturation at 94°C for 30 s, annealing at 55°C for 30 s, and extension at 72°C for 45 s, with a final extension at 72°C for 7 min. All the PCR reactions were performed in the same thermal cycler (Applied Biosystems, Foster, CA, USA). The PCR products were separated by a ABI 3730XL capillary electrophoresis analyzer (Applied Biosystems, Foster, CA, USA) with a GeneScan-500LIZ size standard. Fragments were genotyped for their presence/absence at each locus, and the allele sizes were scored using GeneMaker 2.2.0 software (SoftGenetics LIC, State College, PA, USA) and visually checked twice to reduce genotyping errors. The genotyping error was computed by the blind replication of approximately 13.5% of the amplifications (93 of the 691 samples, evenly distributed among the 25 populations). The repeated genotypes were compared to the previous ones, and the number of allelic differences was counted.

### Data analysis

Null alleles of each locus were checked by MICRO-CHECKER version 2.2.3 (Van Oosterhout et al., 2003), and the number of alleles (*Na*), the effective number of alleles (*Ne*), Shannon's information index (*I*), expected heterozygosity (*He*), observed heterozygosity (*Ho*), and Wright's *F* statistics parameters (*Fis, Fit* and *Fst*) were computed using POPGENE32 software (Yeh et al., 1997). The polymorphic information content (PIC) of each locus was calculated by CERVUS version 3.0 (Kalinowski et al., 2007). An unweighted pair-group method with the arithmetic mean (UPGMA) tree from a matrix of Nei's genetic distances between populations was calculated in POPGENE32 and constructed in MEGA version 5.0 (Tamura et al., 2011). Furthermore, to better investigate the genetic relationships among populations, principal coordinate analysis (PCoA) of the mean pairwise population genetic distance matrix was carried out using the standardized distance method in GenAlEx 6.5 (Peakall and Smouse, 2012). An analysis of molecular variance (AMOVA) among and within populations was carried out using the program Arlequin version 3.5 with 100,000 permutations to ensure the accuracy of the estimation of variance components (Excoffier and Lischer, 2010).

The population genetic structure of 691 individuals was analyzed with the alleles detected by the 11 genic SSR markers using the STRUCTURE 2.3.3 software program (Pritchard et al., 2009) with prior information on the populations (LOCPRIOR model) based on an extended Bayesian cluster analysis. Delta K was developed and tested to prove the real population structure under different simulation routines. Delta K indicated a clear peak at the true value of K. An admixture model was used with 10 iterations per K value ranging from 1 to 10 and assuming correlated allele frequencies, with 1000,000 Markov chain Monte Carlo (MCMC) repetitions after a burn-in period of 100,000. We adopted the height of the Delta K value as an indicator of the strength of the signal detected by the structure analysis (Evanno et al., 2005). For the graphic visualization of the structure results, we used Clustering Markov Packager Across K (CLUMPAK). The Mantel test was performed with Isolation by Distance Web Service 3.2.3 (Jensen et al., 2005) to test the correlation between genetic distance (*Nei's*) and geographic distance (km) to analyze the isolation by distance among populations. The significance was evaluated by performing 10,000 randomization (Mantel, 1967).

## Results

### Characteristics and identification of genic SSRs in *L. formosana* transcriptome data

A total of 80482 unigenes comprising approximately 56.25 Mb in *L. formosana* were searched for the presence of SSRs, and among these unigenes, 9055 unigenes containing 10645 potential SSR loci were detected, with an average frequency of one SSR per approximately 5.28 kb. A total of 10645 potential SSR loci were detected in 9055 unigenes: of which, 7601 (83.94%) carried a single SSR locus and 1454 (16.06%) contained more SSR loci.

The frequencies of different types of genic SSR motifs in *L. formosana* transcriptome data were calculated (Table 2). Approximately 687 motifs were identified, and among all these repeat types, the length of genic SSRs varied from 12 to 60 bp, with an average of 19.71 bp. The dinucleotide was the most abundant repeat unit, accounting for 67.99% of the total genic SSRs, followed by trinucleotide (28.39%), comprising 96.38% by pooling them together. The other repeat units were tetranucleotides (1.95%), pentanucleotides (0.72%), and hexanucleotides (0.94%). The number of SSR motif iterations ranged from 5 to 30, and the most common was *n* = 6 (23.42%), containing the most dinucleotide repeats.

**Table 2:** **Frequencies of different motif types of genic SSRs in *L. formosana* transcriptome data**.

**SSR motif**	**Repeat number**									**Percentage (%)**
	**5**	**6**	**7**	**8**	**9**	**10**	**11**	**>11**	**Total**	
Dinucleotide	0	1685	1043	789	622	553	488	2058	7238	67.99
Trinucleotide	1266	714	441	319	72	85	51	74	3022	28.39
Tetranucleotide	138	52	11	6	0	1	0	0	208	1.95
Pentanucleotide	55	14	5	3	0	0	0	0	77	0.72
Hexanucleotide	54	28	10	4	3	1	0	0	100	0.94
Total	1513	2493	1510	1121	697	640	539	2132	10645	100.00
Percentage (%)	14.21	23.42	14.19	10.53	6.55	6.01	5.06	20.03	100.00	

Of the 72 genic SSR primer pairs designed, 45 pairs successfully produced the expected product by amplification, with a success rate of 62.5%, but only 14 primer pairs showed abundant polymorphisms. The characteristics of the polymorphism primers were presented in Table 3. These results showed that 4 loci were located in the coding sequence (CDS) regions, 4 loci were located in the 5′ untranslated regions (5′-UTR), and the others were located in 3′ untranslated regions (3′-UTR). The majority were located in 5′ and 3′-UTR because the UTR region had more genetic variability than CDS and had more polymorphisms between individuals among populations (Aggarwal et al., 2007).

**Table 3:** **Characteristics of developed genic SSRs**.

**Locus**	**Primer sequence (5′−3′)**	**Repeat motif**	**Position**	**Null alleles**	**GenBank number**
LF 3	F: TGCGAATCACTGGTCGAATCA	(TCT)8	5′-UTR	Yes (1)	KU356822
	R:TCCAACAAGTCAACAACAGCA				
LF 15	F: AGGACCAGCAAGTAACGGTG	(GTG)6	CDS	Yes (17)	KU356823
	R:AGCCATGAAACCGAAGAGCT				
LF 17	F: TCTGGTTATCTCGGGGCAAC	(GCC)6	CDS	NO	KU356824
	R:TGTCAACCAATCTGCCGGAA				
LF 19	F:TAGAACGCCGACTCAAGTGG	(GCA)7	3′-UTR	Yes (2)	KU356825
	R:AAGTTGTTCTGGGCATGGCA				
LF 25	F: ACGGACCCATCTTTACCTGC	(TA)8	5′-UTR	NO	KU356826
	R:TGATACCTCCCTTCTGGCCA	(GA)6			
LF 26	F: ACGGCCTTGGTTTGTTCTGA	(CT)16	5′-UTR	Yes (20)	KU356827
	R:CGACAGATGCAGCTAGGTGT				
LF 29	F: GACAGACCCTCAGAGTTGCC	(AGA)6	3′-UTR	Yes (1)	KU356828
	R:GTTGAACGCCTCTTCTGCTG				
LF 32	F: TGTTCCCACACCATCCTCAC	(CAT)6	CDS	Yes (1)	KU356829
	R:GCCCAGAAGAAGCCAAGTGA				
LF 37	F: TCGCCTCTGTCCTCTCCTAC	(AAC)5	CDS	NO	KU356830
	R:ATGTGCCAGATGTGTTCCGT				
LF 40	F: CCCACCTCAAGCAAGAACCA	(AGA)5	5′-UTR	NO	KU356831
	R:GCCGTGGAGAATGAGAGGTT				
LF 49	F: CCGTTGACATCGCATATCACG	(GGA)5	3′-UTR	Yes (1)	KU356832
	R:TCACTTTCCTATGCTGTCACGA				
LF 62	F: GGTTGCTCTTGTTGGGTCCT	(TGA)7	3′-UTR	NO	KU356833
	R:CAGCCTCACTCAGCCAAGAT				
LF 69	F: AAATAAGCCCTGACGGTGGC	(TGG)6	3′-UTR	Yes (1)	KU356834
	R:GAGACAAAGTGCGGTGGTTG				
LF 72	F: TCGCCTCACTTTTCTAGCGT	(TA)8	3′-UTR	Yes (19)	KU356835
	R: TGCGAAGTCTGACTCGGATG				
Mean	—	—			—

Null alleles: “Yes” means that the null allele was present, “No” means not present; the number in parentheses represents the number of populations, including null alleles.

### Genic SSR polymorphism

MICRO-CHECKER analysis showed that several loci exhibited null alleles in some populations. At LF 15, LF 26, LF 72, null alleles were detected in the 17, 20, and 19 populations, respectively (Table 3). So, these three loci were deleted in later analyses. At LF 3, LF 29, LF 32, LF 49 and LF 69, null alleles were detected in only one population; at LF 19, null alleles were detected in two populations; nevertheless, no null alleles were detected in the rest of loci. A total of 42 allelic differences were found in the 2046 alleles examined, and the genotyping error rate was estimated to be 2%. Therefore, these suggested that 11 SSR loci were effective to assess the genetic diversity and population structure of *L. formosana*.

A total of 67 alleles were generated by the 11 SSR loci in all samples. The number of alleles (*Na*) per locus ranged from 3 (LF 40) to 10 (LF 69), with a mean of 6.0909, whereas the effective number of alleles (*Ne*) per locus varied from 1.2709 (LF 40) to 2.8047 (LF 17), with an average value of 1.9266. The Shannon's Information Index (*I*) had an average of 0.8178 and ranged from 0.3823 (LF 40) to 1.2657 (LF 19). Observed heterozygosity (*Ho*) and expected heterozygosity (*He*) varied from 0.2012 (LF 40) to 0.6411 (LF 17), and 0.2133 (LF 40) to 0.6439 (LF 17), with an average of 0.4090 and 0.4322, respectively. *He* was higher than *Ho* at 10 loci, with the exception being LF 25, in accordance with the mean inbreeding coefficient at the total population (*Fit* = 0.056). The value of the polymorphism information content (*PIC*) ranged from 0.192 (LF 40) to 0.580 (LF 17 and LF 19) with a mean of 0.3904 (Table 4). The value of *PIC* showed that highly polymorphic loci (*PIC* > 0.5) comprised 36.4% of all loci, whereas moderately polymorphic (0.25 < *PIC* < 0.5), and lowly polymorphic (*PIC* < 0.25) loci occupied 36.4% and 27.2%, respectively.

**Table 4:** **Polymorphism analysis of SSR primers in 11 loci**.

**Locus**	***Na***	***Ne***	***I***	***Ho***	***He***	***PIC***	***Fis***	***Fit***	***Fst***
LF 3	5	1.7979	0.8224	0.4023	0.4441	0.401	0.0277	0.0961	0.0704
LF 17	7	2.8047	1.1785	0.6411	0.6439	0.58	−0.0623	0.0026	0.061
LF 19	7	2.5907	1.2657	0.5658	0.6144	0.58	−0.0185	0.0821	0.0987
LF 25	9	2.1646	1.0111	0.6136	0.5384	0.468	−0.1915	−0.1354	0.047
LF 29	5	1.5803	0.623	0.3386	0.3675	0.312	0.0413	0.0866	0.0472
LF 32	6	1.2715	0.3927	0.1983	0.2137	0.193	0.025	0.0747	0.051
LF 37	6	2.3942	1.057	0.547	0.5827	0.521	−0.0044	0.0622	0.0663
LF 40	3	1.2709	0.3823	0.2012	0.2133	0.192	0.0005	0.0465	0.0461
LF 49	5	1.404	0.6187	0.259	0.2879	0.273	0.0386	0.1059	0.07
LF 62	4	1.299	0.4172	0.2214	0.2303	0.206	−0.0567	0.0328	0.0847
LF 69	10	2.6151	1.227	0.5109	0.618	0.567	0.0476	0.1818	0.1409
Mean	6.0909	1.9266	0.8178	0.409	0.4322	0.3904	−0.0213	0.056	0.0757

Na, Number of alleles observed; Ne, Effective number of alleles; I, Shannon's information index; Ho, Observed heterozygosity; He, Expected heterozygosity. Fis, Inbreeding coefficient at the population level; Fit, Inbreeding coefficient at total populations; Fst, Proportion of differentiation among populations.

The genetic diversity at the population and region level is presented in Table 5. The *Na* and *Ne* per population ranged from 2.727 to 3.636 and from 1.597 to 2.151, respectively. The *Ho* and *He* varied from 0.282 to 0.496 and from 0.302 to 0.469, respectively. In addition, private alleles were found in the KX, XY, TR, TB, HSAH, KH, PX, and TG populations, suggesting that much greater special genetic variation was presented in these natural geographic populations. Expected heterozygosity is a significant measurement of the genetic diversity of populations (Slatkin and Barton, 1989). At the population level, the highest genetic diversity was found in population XY (*He* = 0.469), whereas the lowest was detected in population BWL (*He* = 0.302). In population SZHN, *He* (0.341) was higher than *Ho* (0.287), which implied that there was a deficit of heterozygosity in the population, possibly due to the existence of inbreeding. We assessed the level of genetic variation among four different regions, and these results showed that the highest genetic diversity (*He* = 0.435, *I* = 0.760) and the largest number of private alleles (*n* = 5) was found in the Southwestern populations, whereas the lowest genetic diversity (*He* = 0.358, *I* = 0.644) and a relatively low number of private alleles (*n* = 1) were discovered in the Central populations.

**Table 5:** **Genetic diversity of 25 populations of *L. formosana* revealed by SSRs**.

**Region**	**Population**	***Na***	***Ne***	***Ho***	***He***	***I***	**Private alleles**
Southwestern	KX	3.364	2.151	0.451	0.459	0.810	1
	LY	2.727	2.075	0.407	0.443	0.737	0
	GY	3.091	1.953	0.448	0.434	0.742	0
	FD	3.364	1.915	0.496	0.436	0.756	0
	XY	3.545	2.108	0.474	0.469	0.845	1
	TR	3.545	1.852	0.421	0.413	0.741	3
	CB	3.273	1.965	0.437	0.439	0.779	0
	FN	3.000	1.774	0.423	0.390	0.670	0
	Mean	3.239	1.974	0.445	0.435	0.760	—
Dabie Mountains/Foothills	SC	3.364	1.719	0.406	0.386	0.676	0
	TB	3.182	1.900	0.471	0.428	0.736	1
	HA	3.364	1.722	0.424	0.374	0.678	0
	Huos	3.364	1.750	0.408	0.383	0.705	0
	HSAH	3.636	1.973	0.427	0.413	0.769	1
	WYJX	3.273	1.964	0.395	0.414	0.751	0
	KH	3.182	1.680	0.372	0.340	0.633	1
	Mean	3.338	1.815	0.415	0.391	0.707	—
Coastal	NJ	3.364	1.912	0.447	0.432	0.767	0
	ZS	3.545	1.962	0.409	0.414	0.758	0
	JO	3.273	1.851	0.470	0.432	0.765	0
	CX	3.000	1.666	0.332	0.338	0.621	0
	PX	3.455	1.872	0.400	0.409	0.737	1
	BWL	3.091	1.597	0.282	0.302	0.563	0
	Mean	3.288	1.810	0.390	0.388	0.702	—
Central	WYGD	3.182	1.728	0.364	0.361	0.656	0
	SZHB	3.000	1.802	0.370	0.381	0.664	0
	SZHN	3.273	1.658	0.287	0.341	0.645	0
	TG	3.000	1.653	0.370	0.347	0.611	1
	Mean	3.114	1.710	0.348	0.358	0.644	—
	Overall mean	3.258	1.848	0.408	0.399	0.713	—

### Genetic differentiation and genetic structure

The inbreeding coefficient within populations (*Fis*) per locus varied from −0.1915 (LF 25) to 0.0476 (LF 69) with a mean of −0.0213, and these results indicated an excess of heterozygosity. However, a deficiency of heterozygosity was discovered at six loci: LF 3, LF 29, LF 32, LF 40, LF 49, and LF 69. Furthermore, the inbreeding coefficient at total populations (*Fit*) ranged from −0.1354 (LF 25) to 0.1818 (LF 69) with an average of 0.056. Moreover, the genetic differentiation (*Fst*) ranged from 0.0461 (LF 40) to 0.1409 (LF 69) with a mean of 0.0757, which indicated moderate differentiation among populations (Table 4). Analysis of molecular variance (AMOVA) was adopted to evaluate the diversity of components within and among populations. These results indicated that the genetic variation mainly occurred within populations, accounting for 94.02% of the total variation, whereas the genetic variation among populations was only 5.98% (Table 6).

**Table 6:** **Analysis of molecular variance (AMOVA) of genetic diversity of *L. formosana* populations**.

**Source of variation**	**d.f**.	**Sum of squares**	**Variance components**	**Percentage of variation (%)**	***P***
Among populations	24	247.817	0.14259	5.98	< 0.001
within populations	1357	3040.099	2.24031	94.02	< 0.001
Total	1381	3382.916	2.38290		

**Figure 2:**
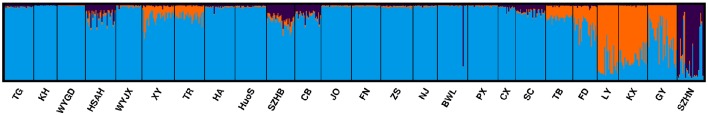
**Inferred population structure of *L. formosana* in China using program STRUCTURE (*K* = 3)**. Each individual is represented by a single vertical line, which is partitioned into colored segments in proportion to the estimated membership in 3 groups: blue for Group 1, orange for Group 2, and purple for Group 3.

The statistical model described by Evanno et al. (2005) indicated that the highest peak (Delta *K* = 16.13) was at the value *K* = 3 (Supplementary Figure 1). STRUCTURE analysis showed that *K* = 3 was the optimum number of subpopulations, revealing that at least three distinct groups existed among 25 populations (Figure 2). These results suggested that there were different degrees of introgression among populations.

### Genetic relationships and patterns of geographic variation

**Figure 3:**
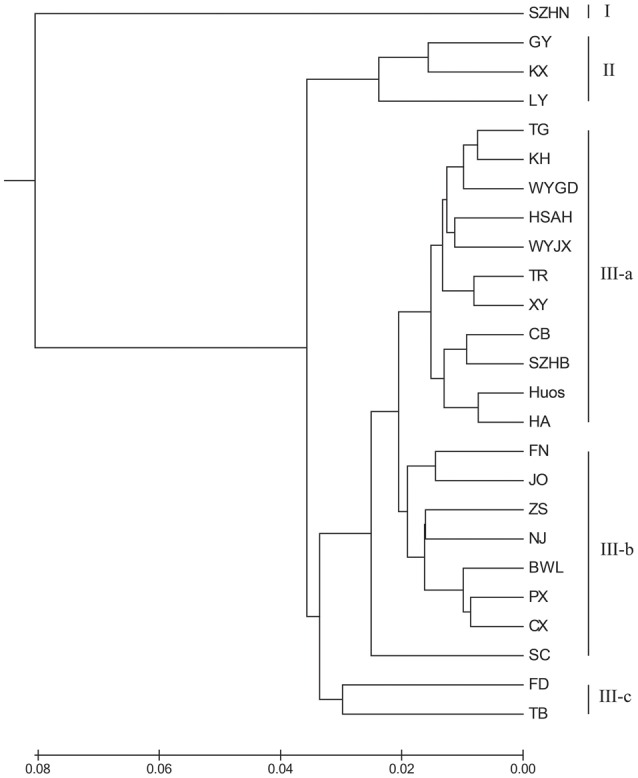
**UPGMA dendrogram showing the relationships of *L. formosana* populations in China based on genetic distance**.

**Figure 4:**
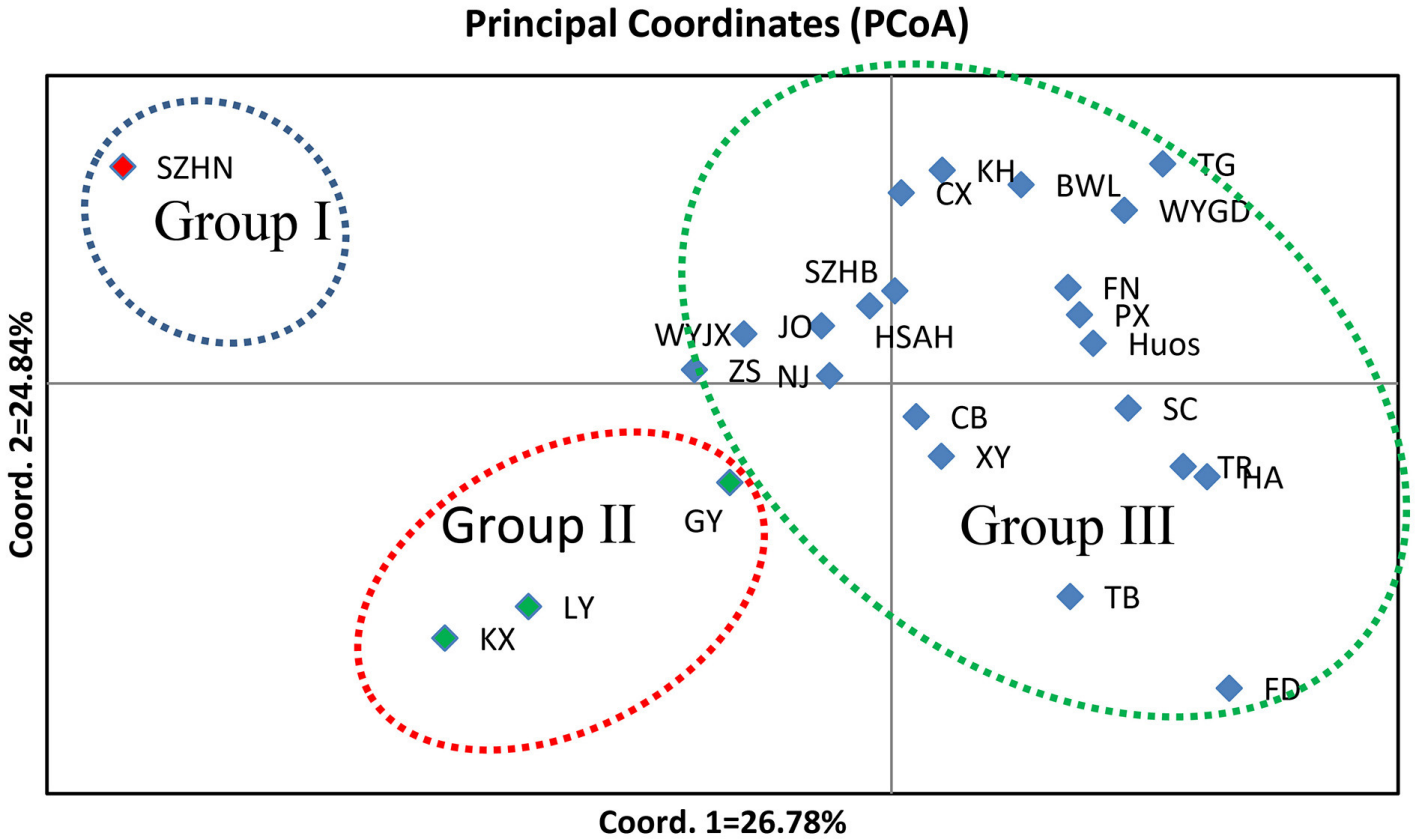
**A principal coordinate analysis (PCoA) based on pairwise genetic distance estimates for all populations**. Percentages of total variance explained by coordinate 1 and 2 accounting for 26.78 and 24.84%, respectively.

To illustrate the genetic relationships among the 25 populations, a UPGMA dendrogram (Figure 3) was constructed based on the Nei's genetic distances (Supplementary Table 1). The dendrogram showed that these populations were grouped into three clusters, which are essentially identical to those determined by PCoA analysis (Figure 4). Cluster I only involved population SZHN, cluster II included populations of LY, KX, and GY, the three populations located in the south of Qinling Mountains, and the rest were classified as cluster III. Cluster III was further separated into three sub-clusters of III-a, III-b and III-c. Populations of III-a were mainly located in Southwestern China and the Dabie Mountains and Foothills regions; Populations of III-b were mainly situated in the Coastal regions; III-c contained TB and FD populations. These results suggested that there was no distinct geographical structure among populations. Populations from geographically close locations did not cluster together, other than populations KX, GY, and LY in Cluster II.

The Mantel test revealed that no correlation was found between genetic distance and geographic distance in *L. formosana* (*r* = 0.0500, *P* = 0.6940). These results provided further evidence that geographic isolation was not the primary factor leading to moderate genetic differentiation of *L. formosana.*

## Discussion

### Development of genic SSR markers in *L. formosana*

The mean frequency of SSR loci detected in *L. formosana* was one SSR locus per 5.28 kb, which is lower than that of *Larix gmelinii* (one SSR locus per 2.87 kb) (Zhang et al., 2015), but higher than that of *Pinus dabeshanensis* (one SSR locus per 23.08 kb) (Xiang et al., 2015). These differences may be caused by the application of different repeat unit criteria and repeat lengths, the number of databases searched, and SSR identification tools (Dutta et al., 2011). Compared with some previous studies, these results suggested that dinucleotide motifs are the most common genic repeat units in most species, such as rubber (Li et al., 2012), and grape (Scott et al., 2000). The genic SSRs located in coding sequence (CDS) regions were all trinucleotide repeats, and variation was present at the higher taxonomic levels. In the same way, these CDS SSRs are also highly transferable to other species of the genus *Liquidambar*. The availability of a selection of the SSR coding structure (UTRs or CDSs) may be helpful for targeting genic SSRs of plant materials at different taxonomic levels.

### Genetic diversity in *L. formosana*

Chinese sweetgum is characterized by its wide range of distribution, large height and diameter, wind-pollination, outcrossing and self-incompatible mating system, allowing the species to develop a higher genetic diversity. Surprisingly, the overall genetic diversity (*He* = 0.399) detected in our study was only relatively moderate. The genetic diversity was lower than the average value (*He* = 0.650) of outcrossing plants using SSR markers (Nybom, 2004). Moreover, a similar phenomenon was also observed in cpDNA markers in previous studies of Chinese sweetgum (Wu, 2009). These may be attributed to historical evolution events and severe climatic changes, particularly Quaternary repetitive glaciations (Li, 1975). The genetic diversity of sweetgum populations would have been erased by repetitive glacial contraction and expansion, and the founder effect of the population would lead to a reduction in genetic diversity. We speculated that population BWL could have experienced the founder effect because the island of Hainan was connected to the mainland due to lower sea levels during the Pleistocene (Hsu, 1983), allowing repetitive gene exchange between the mainland populations and the island population. However, genetic diversity declined when the link was broken.

In general, outcrossing and a self-incompatible mating system will produce a remarkable level of within-population genetic variation (Hamrick and Godt, 1989). In our study, the average value of *Fst* was 0.0757, which stands for a moderate level of population differentiation. This was also shown in the AMOVA, which indicated that only 5.98% of variation was attributable to population diversity. Furthermore, the Mantel test displayed a lack of correlation between genetic variation and geographic isolation in the sampled populations. Moderate levels of total genetic differentiation were also reported at the population level in Chinese sweetgum using ISSR markers; 14.51% of the genetic variation was found within populations, and 85.49% was found among populations (Bi et al., 2010). High gene flow could be the cause of moderate genetic differentiation. High gene flow may have resulted from long distance gene dispersal by pollen or small winged seeds (Yao et al., 2007). We suggest that long distance pollen dispersal may be the foremost evolutionary force that influences the genetic structure in sweetgum, and this is line with a previous study in *L. styraciflua* of the same genus (Nuttle and Haefner, 2005).

### Population relationships and structure in *L. formosana*

The results indicated that the 25 populations under study were grouped into three clusters (Figure 3). Cluster III was an important and diverse group, which was mainly distributed in Southwestern China, the Coastal region and Dabie Mountains and adjacent regions. These regions extend across tropical and subtropical zones and hosted most of the private alleles. Although the TB and FD populations were far apart geographically, they were clustered together in the III-c, and these two populations had relatively higher genetic diversity. Moreover, populations from the northern margin of distribution in Cluster II were mainly located in southern area of the Qinling Mountains. It should be noted that Cluster I (population SZHN), situated in the center of the distribution, was obviously separate from other populations in the UPGMA cluster dendrogram. The Southwestern region displayed the highest genetic diversity, followed by the Dabie Mountains and Foothills and Coastal regions, whereas the Central region showed the lowest genetic diversity and deficit of heterozygosity. Compared with the genetic diversity at the region level, we speculated that Southwestern China may be the center of genetic diversity for this species, and the lower genetic diversity of the marginal population could be the result of geographic isolation or the founder effect.

Given the above information, Southwestern China may be the glacial refugia for Chinese sweetgum due to the mountain ranges extending from east to west. The post-glacial dispersal of the species was largely based on Southwestern China and likely experienced biotic interventions in the process of dispersal, although additional chloroplast or mitochondria data are needed to assess this hypothesis.

### Implications for the management and conservation of genetic resources

Our analyses proved the usefulness of SSR markers to provide deep insight into the genetic background of *L. formosana*. The results indicated that moderate genetic diversity and genetic differentiation of *L. formosana* was found in widespread areas. However, during the period of China's self-reliance on steel, extensive deforestation destroyed the ecological environment of *L. formosana*. In the field investigation, we found that many natural individuals of *L. formosana* were destroyed to plant other tea trees. Therefore, the focus of the conservation of genetic resources should be on preventing further direct damage to the existing populations and collecting genetic resources from the center of genetically diverse populations as well as from peripheral populations for complementary *ex situ* conservation measures. Moreover, the highest genetic diversity population XY could be given the highest priority for the protection of the original habitat. For *ex situ* conservation, Southwestern populations could also serve as seed sources, given their high level of genetic variability. The presence of private alleles in the natural population also provides opportunities for selective breeding for greater adaptation and higher resistance to changing environments. In addition, as most of the genetic diversity of *L. formosana* exists among individuals within a population, individual plant selection would be an effective way of using natural variations in genetic improvement programs. It is critical to develop a strategy for the conservation and breeding of *L. formosana* that is not only helpful in protecting genetic resources but also in attaining effective management and exploiting genetic resources.

## Author contributions

RS, FL, and YZ conceived and designed the experiments; RS and FL collected plant materials; RS performed the experiments and drafted the manuscript; YZ, FL, and PH edited the manuscript. All authors read and approved the manuscript.

## Funding

This study was supported by the project of Gene Discovery and Innovative Use of Forest Genetic Resources (2013BAD01B06) and the National Forest Genetic Resource Platform (2005DKA21003).

### Conflict of interest statement

The authors declare that the research was conducted in the absence of any commercial or financial relationships that could be construed as a potential conflict of interest.
