# Supplementary material for: Moderate Genetic Diversity and Genetic Differentiation in the Relict Tree Liquidambar formosana Hance Revealed by Genic Simple Sequence Repeat Markers
Source: Front Plant Sci. 2016 Sep 21;7:1411. doi: 10.3389/fpls.2016.01411 (PMC5030344; doi:10.3389/fpls.2016.01411)
Supplement: Supplementary file 1 [file Table1.docx]

Supplementary Material

**Moderate Genetic Diversity and Genetic Differentiation in the Relict**

**Tree *Liquidambar formosana* Hance Revealed by Genic Simple**

**Sequence Repeat Markers**

***Rongxi Sun ^†^, Furong Lin ^†^, Ping Huang and Yongqi Zheng****

*** Correspondence:** *Yongqi Zheng*: [zyq8565@126.com](mailto:zyq8565@126.com)

^1^ These authors contributed equally to this work.

# Supplementary Figures and Tables

For more information on Supplementary Material and for details on the different file types accepted, please see [here](http://home.frontiersin.org/about/author-guidelines#SupplementaryMaterial).

## Supplementary Figures

**Supplementary Figure 1.** The log likelihood of the data ((Delta K) as a reference K over ten iterations

## Supplementary Tables

**Supplementary Table 1.**

Nei's genetic distances among *L. formosana* populations

|  | **BWL** | **PX** | **TR** | **XY** | **HSAH** | **KX** | **LY** | **TG** | **WYJX** | **CB** | **KH** | **WYGD** | **FN** | **FD** | **JO** | **ZS** | **SZHN** | **SZHB** | **NJ** | **GY** | **CX** | **Huos** | **SC** | **TB** | **HA** |
| --- | --- | --- | --- | --- | --- | --- | --- | --- | --- | --- | --- | --- | --- | --- | --- | --- | --- | --- | --- | --- | --- | --- | --- | --- | --- |
| **BWL** | 0.000 |  |  |  |  |  |  |  |  |  |  |  |  |  |  |  |  |  |  |  |  |  |  |  |  |
| **PX** | 0.022 | 0.000 |  |  |  |  |  |  |  |  |  |  |  |  |  |  |  |  |  |  |  |  |  |  |  |
| **TR** | 0.068 | 0.037 | 0.000 |  |  |  |  |  |  |  |  |  |  |  |  |  |  |  |  |  |  |  |  |  |  |
| **XY** | 0.048 | 0.027 | 0.016 | 0.000 |  |  |  |  |  |  |  |  |  |  |  |  |  |  |  |  |  |  |  |  |  |
| **HSAH** | 0.057 | 0.047 | 0.032 | 0.020 | 0.000 |  |  |  |  |  |  |  |  |  |  |  |  |  |  |  |  |  |  |  |  |
| **KX** | 0.114 | 0.088 | 0.049 | 0.040 | 0.057 | 0.000 |  |  |  |  |  |  |  |  |  |  |  |  |  |  |  |  |  |  |  |
| **LY** | 0.135 | 0.119 | 0.087 | 0.070 | 0.083 | 0.050 | 0.000 |  |  |  |  |  |  |  |  |  |  |  |  |  |  |  |  |  |  |
| **TG** | 0.052 | 0.047 | 0.024 | 0.027 | 0.024 | 0.068 | 0.110 | 0.000 |  |  |  |  |  |  |  |  |  |  |  |  |  |  |  |  |  |
| **WYJX** | 0.066 | 0.060 | 0.039 | 0.020 | 0.023 | 0.054 | 0.071 | 0.029 | 0.000 |  |  |  |  |  |  |  |  |  |  |  |  |  |  |  |  |
| **CB** | 0.063 | 0.042 | 0.029 | 0.023 | 0.029 | 0.052 | 0.086 | 0.028 | 0.033 | 0.000 |  |  |  |  |  |  |  |  |  |  |  |  |  |  |  |
| **KH** | 0.046 | 0.039 | 0.028 | 0.025 | 0.018 | 0.058 | 0.093 | 0.015 | 0.021 | 0.025 | 0.000 |  |  |  |  |  |  |  |  |  |  |  |  |  |  |
| **WYGD** | 0.033 | 0.027 | 0.027 | 0.022 | 0.030 | 0.082 | 0.097 | 0.020 | 0.029 | 0.036 | 0.020 | 0.000 |  |  |  |  |  |  |  |  |  |  |  |  |  |
| **FN** | 0.043 | 0.033 | 0.036 | 0.022 | 0.039 | 0.063 | 0.074 | 0.029 | 0.038 | 0.028 | 0.033 | 0.023 | 0.000 |  |  |  |  |  |  |  |  |  |  |  |  |
| **FD** | 0.109 | 0.070 | 0.031 | 0.043 | 0.057 | 0.069 | 0.112 | 0.061 | 0.077 | 0.061 | 0.072 | 0.068 | 0.079 | 0.000 |  |  |  |  |  |  |  |  |  |  |  |
| **JO** | 0.040 | 0.034 | 0.065 | 0.046 | 0.052 | 0.094 | 0.092 | 0.053 | 0.049 | 0.044 | 0.042 | 0.039 | 0.029 | 0.104 | 0.000 |  |  |  |  |  |  |  |  |  |  |
| **ZS** | 0.031 | 0.040 | 0.063 | 0.048 | 0.046 | 0.067 | 0.107 | 0.046 | 0.057 | 0.047 | 0.037 | 0.050 | 0.055 | 0.089 | 0.041 | 0.000 |  |  |  |  |  |  |  |  |  |
| **SZHN** | 0.187 | 0.187 | 0.177 | 0.143 | 0.097 | 0.165 | 0.190 | 0.155 | 0.147 | 0.103 | 0.126 | 0.176 | 0.157 | 0.226 | 0.183 | 0.158 | 0.000 |  |  |  |  |  |  |  |  |
| **SZHB** | 0.051 | 0.044 | 0.031 | 0.028 | 0.028 | 0.059 | 0.080 | 0.032 | 0.045 | 0.019 | 0.025 | 0.030 | 0.025 | 0.072 | 0.045 | 0.047 | 0.095 | 0.000 |  |  |  |  |  |  |  |
| **NJ** | 0.040 | 0.026 | 0.047 | 0.026 | 0.032 | 0.071 | 0.104 | 0.040 | 0.037 | 0.041 | 0.030 | 0.036 | 0.043 | 0.058 | 0.040 | 0.032 | 0.156 | 0.049 | 0.000 |  |  |  |  |  |  |
| **GY** | 0.065 | 0.045 | 0.037 | 0.030 | 0.048 | 0.031 | 0.046 | 0.055 | 0.047 | 0.047 | 0.043 | 0.043 | 0.034 | 0.069 | 0.045 | 0.058 | 0.183 | 0.037 | 0.048 | 0.000 |  |  |  |  |  |
| **CX** | 0.018 | 0.017 | 0.042 | 0.032 | 0.039 | 0.076 | 0.103 | 0.034 | 0.042 | 0.034 | 0.020 | 0.026 | 0.026 | 0.099 | 0.026 | 0.027 | 0.154 | 0.031 | 0.031 | 0.042 | 0.000 |  |  |  |  |
| **Huos** | 0.035 | 0.026 | 0.019 | 0.024 | 0.022 | 0.058 | 0.097 | 0.017 | 0.036 | 0.024 | 0.015 | 0.024 | 0.030 | 0.055 | 0.033 | 0.029 | 0.156 | 0.024 | 0.032 | 0.033 | 0.016 | 0.000 |  |  |  |
| **SC** | 0.042 | 0.051 | 0.047 | 0.046 | 0.057 | 0.088 | 0.108 | 0.056 | 0.078 | 0.048 | 0.056 | 0.053 | 0.043 | 0.068 | 0.051 | 0.053 | 0.172 | 0.031 | 0.071 | 0.054 | 0.050 | 0.033 | 0.000 |  |  |
| **TB** | 0.105 | 0.056 | 0.042 | 0.054 | 0.071 | 0.076 | 0.110 | 0.068 | 0.078 | 0.048 | 0.063 | 0.061 | 0.063 | 0.060 | 0.060 | 0.085 | 0.204 | 0.058 | 0.051 | 0.052 | 0.068 | 0.047 | 0.092 | 0.000 |  |
| **HA** | 0.053 | 0.043 | 0.028 | 0.039 | 0.039 | 0.079 | 0.108 | 0.035 | 0.057 | 0.025 | 0.033 | 0.041 | 0.043 | 0.053 | 0.048 | 0.047 | 0.166 | 0.032 | 0.049 | 0.058 | 0.033 | 0.015 | 0.036 | 0.051 | 0.000 |
