# Supplementary figures and images for: Moderate Genetic Diversity and Genetic Differentiation in the Relict Tree Liquidambar formosana Hance Revealed by Genic Simple Sequence Repeat Markers
Source: Front Plant Sci. 2016 Sep 21;7:1411. doi: 10.3389/fpls.2016.01411 (PMC5030344; doi:10.3389/fpls.2016.01411)

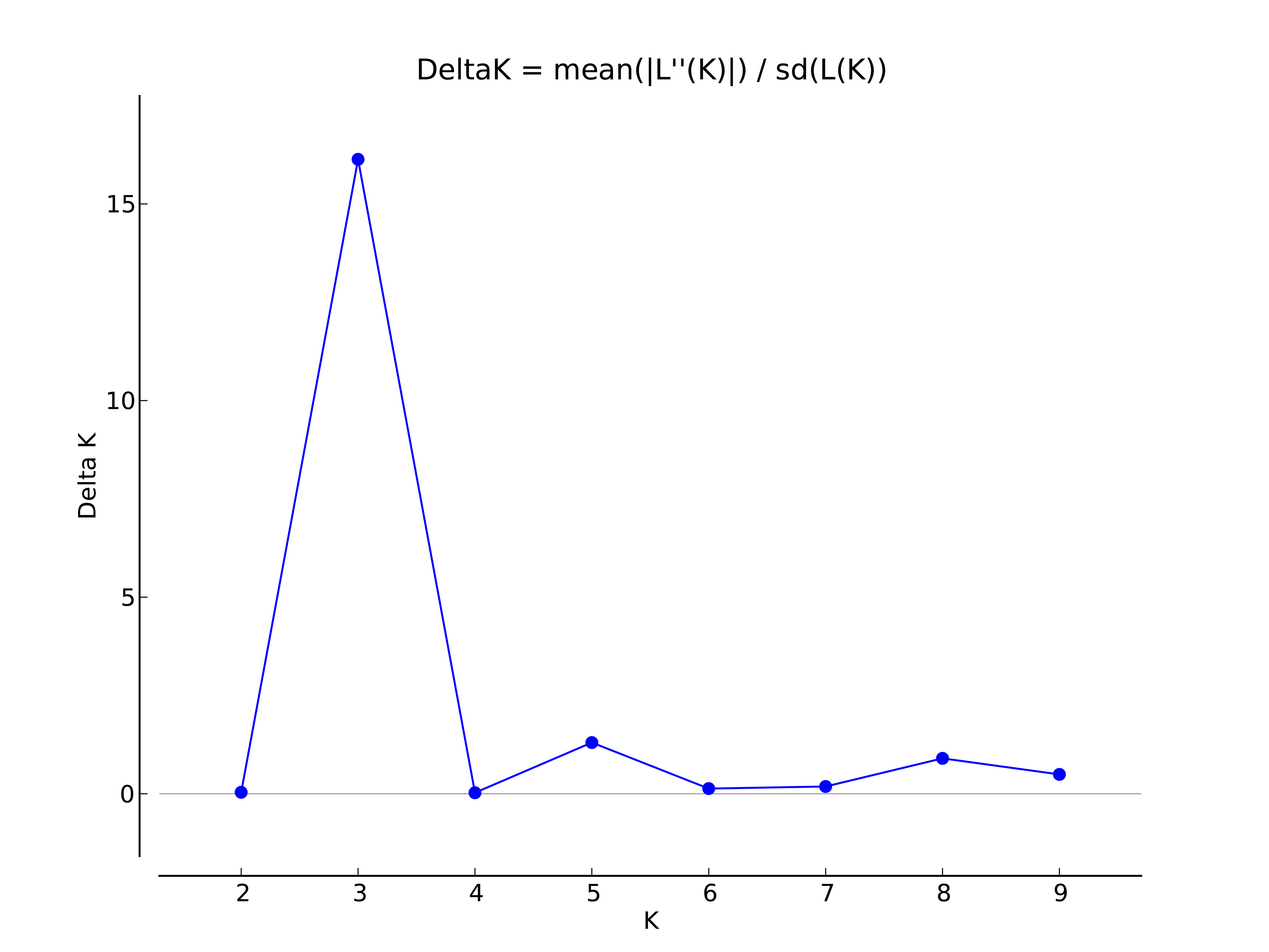

Supplement: Supplementary file 2 [file Image1.JPEG]
